# Acetate of Ammonia in Uterine Diseases

**Published:** 1829-03

**Authors:** 


17. *Acetate of Ammonia in Uterine Diseases.*—Few diseases, not fatal in their tendency, prove more obstinate, and inflict greater pain, than dysmenorrhœa; while its general effect, sterility, gives to it a distinct quality of evil. Every physician must have been disappointed in the effects of the remedies which are in common use for this malady, and be prepared, therefore, to adopt any new recipe.

Sometime since Mr. Cloquet, of Paris, published an account of a case successfully treated with acetate of ammonia. Subsequently Professor Mazuyer of Strasburg, presented the profession with a favourable report of his trials with the same medicine. More lately Dr. Patin, in the Arch. Gen. de Med. has made a publication on the powers of the acetate in several different forms of uterine disease. His memoir concludes with an enumeration of the diseases of the uterus, to which he considers the acetate applicable:

1st. To cases of painful menstruation, though some degree of care is required in its employment, since it diminishes the quantity of the discharge.

2d. To cases of profuse catamenia and uterine hemorrhagies, affections in which he has obtained the most remarkable results.

3d. To cancers of the uterus, in which disease it will prove at least a powerful palliative.

4th. To nymphomania. Its supposed efficacy in this affection rests upon its influence in case fifth.

It is also recommended in threatened abortion, when this accident is constitutional, and depends upon a too great determination of blood to the uterus; in inflammation of uterus and ovaries; in the different organic lesions of these parts; in a word, in every case in which there exists sur-excitation of the genital system of the female. Care, it is stated, will be required, lest the uterine action be reduced below its natural standard.

Before meeting with the report of Mr. Cloquet, we had used a solution of the acetate of ammonia, as a vehicle for the compound tincture of guaiac, in a case of dysmenorrhœa; and thought the prescription more beneficial, than when the tincture was given in wine, milk, and the other liquids generally employed to dilute it.

It should be recollected that the old fashioned Spiritus Mindereri, is substantially a solution of the acetate of ammonia, and may be substituted for the officinal preparation by those who cannot conveniently obtain the latter.

